# Tislelizumab combined with gemcitabine as first-line treatment in cisplatin-ineligible patients with locally advanced or metastatic urothelial carcinoma: a single center, single-arm phase 2 trial

**DOI:** 10.3389/fimmu.2026.1824893

**Published:** 2026-06-08

**Authors:** Mengyao Liu, Zengjun Liu, Jing Xu, Xin Xu, Qibing Wu, Dongyuan Zhu

**Affiliations:** 1 Department of Oncology, The First Affiliated Hospital of Anhui Medical University, Hefei, Anhui, China; 2 Rare Tumors Department, Shandong Cancer Hospital and Institute, Shandong First Medical University and Shandong Academy of Medical Sciences, Huaiyin, Jinan, China; 3 Department of Oncology, the First Affiliated Hospital of Anhui Medical University, Hefei, China

**Keywords:** cisplatin-ineligible, combine therapy, gemcitabine, tislelizumab, urothelial carcinoma, combination therapy

## Abstract

**Introduction:**

Patients with la/mUC have an unfavorable prognosis and limited treatment options, particularly those who were considered ineligible for cisplatin-containing therapeutic schedule. ICIs have played a significant therapeutic role among this group of patients. Ongoing clinical investigations continue to explore the utility of ICIs, both used alone or in combination with other treatment methods.

**Methods:**

The purpose of this clinical trial is to explore the efficacy and safety of gemcitabine combined with tislelizumab as initial treatment for the la/mUC patients who are intolerant to platinum-based drugs. Primary endpoints were ORR and DCR. Secondary endpoints comprised PFS and OS.

**Results:**

At the data cutoff (September 1, 2025), 30 patients were enrolled. As the results display, the mPFS was 13.9 months (95% CI: 11.4–16.3), and the mOS was 23.3 months ([IQR]: 14.4–33.1; 95% CI: 18.2–28.3).The confirmed ORR was 46.7% (14/30; 95% CI: 28.3–65.7), DCR was 76.7%. The most frequent TRAEs were hematological toxicities, including leukopenia (43.3%) and neutropenia (40.0%). We also explored the predictive potential of tumor-associated TLS as an innovative analysis. There was a quantitative difference in ORR according to the results of TLS analysis, with response rates of 66.7% (n=9) in TLS positive group and 35.3% (n=17) in negative group. However, this observed change did not reach statistical significance (P = 0.218).

**Discussion:**

In conclusion, the combination of tislelizumab and gemcitabine demonstrated manageable tolerability and a promising preliminary efficacy signal as first-line therapeutic strategy for la/mUC patients who cannot receive cisplatin-containing regimen and warrants further investigation.

**Clinical Trial Registration:**

https://www.chictr.org.cn/index.html, identifier ChiCTR2200061631.

## 1 Introduction

Urothelial carcinoma (UC) represents a malignant neoplasm that arises within the urinary tract, with potential origins in the bladder, renal pelvis, or ureter. Sensitive surveillance methods are of great importance for these patients, for it is one of tumors harboring highest mutational burdens (1). Urothelial bladder cancer represents the predominant subtype, comprising over 90% of all bladder cancer cases (2). Nevertheless, in Eastern Asian countries, upper tract urothelial carcinoma (UTUC) demonstrates a significantly elevated prevalence, constituting more than 30% of UC instances (3–5).

Locally advanced or metastatic urothelial carcinoma (la/mUC) is an aggressive malignant tumor which primarily occurs in elderly individuals, and is related to a poor prognosis and high mortality rate. The standard first-line therapeutic approach typically employs chemotherapy regimens centered around cisplatin. However, approximately 50% of patients diagnosed with this condition are considered ineligible for cisplatin therapy owing to compromised renal function, suboptimal performance status, or the presence of concurrent comorbidities (6). Specifically, individuals with UTUC may face particular ineligibility owing to diminished renal function following radical nephroureterectomy (7).

An alternative therapeutic approach to cisplatin-based chemotherapy involves the combination of carboplatin and gemcitabine. Nevertheless, this regimen demonstrates limited efficacy and is frequently associated with poor tolerability, as evidenced by an objective response rate (ORR) of 36% and a median overall survival (OS) duration of 9.3 months. Furthermore, the correlated adverse events pose substantial challenges for elderly and frail patients (8, 9).

The appearance of immunotherapy has introduced novel treatment avenues for urothelial carcinoma, particularly for cisplatin-intolerant patients. The U.S. Food and Drug Administration (FDA) has sanctioned the use of PD-1/PD-L1 inhibitors as a first-line therapeutic modality for patients who exhibit high PD-L1 expression determined by immunohistochemistry. Some regions also approve these agents for patients who are ineligible for platinum chemotherapy, owing to the favorable safety and tolerability profile compared to platinum regimens. Although PD-1/PD-L1 inhibitors can produce durable responses, objective tumor regression is observed in only approximately 20–30% of patients not selected by PD-L1 expression (10, 11). Consequently, the clinical use of them is increasingly being confined to specific subgroups.

In recent years, the advent of antibody-drug conjugates (ADCs), exemplified by enfortumab vedotin (EV), has significantly transformed the therapeutic paradigm for patients suffering from la/mUC, irrespective of their tolerance or intolerance to platinum-based therapies (12, 13). While the combination of EV and pembrolizumab represents the preferred first-line standard in platinum ineligible patients, in many regions, due to limitations in accessibility and cost, as well as the impact of side effects, exploring alternative chemotherapy-immunotherapy strategies is imperative.

Tertiary lymphoid structures (TLSs) are an ectopic lymphoid tissue involved in chronic inflammation and autoimmune responses. TLS has been found in most solid tumors of the urinary system. The progression of tumors induces cascade reaction by releasing multiple cytokines which can promote the gathering of immune cells and the formation of TLSs, thereby regulating tumor growth. The composition of TLSs includes B cells in the middle, surrounded by T cells with characteristic high endothelial venules (HEVs). Fibroblasts and fibroblastic reticular cells accumulate at the tumor junction, while dendritic cells and follicular dendritic cells are scattered throughout. Recent investigations have demonstrated that TLS within cancerous lesions are indicative of enhanced prognostic outcomes (14) and correlate with responsiveness to ICI-based therapeutic interventions (15–17). In this study, we explored TLS as an exploratory biomarker candidate for evaluating treatment efficacy.

This investigation presents the results of a phase 2, single-center, single-arm clinical trial aimed at evaluating the safety profile and therapeutic effectiveness of Tislelizumab in conjunction with gemcitabine as a first-line intervention for patients with la/mUC who are ineligible for cisplatin-based therapy. At present, this is the first study recording the application of this therapeutic strategy in this specific patient population to the best of our knowledge, and the promising findings are anticipated to offer appropriate therapeutic alternatives for this subset of patients. Enrollment for the trial has been finalized and associated data analyses have been completed. The trial was registered on ClinicalTrials.gov under the identifier ChiCTR2200061631 on June 29th, 2022.

## 2 Methods

### 2.1 Research framework and enrolled cohort

This study was an exploratory study with a fixed sample and was designed to evaluate the efficacy of tislelizumab plus gemcitabine as an initial treatment strategy for patients with la/mUC who are ineligible for cisplatin-based therapy. The treatment was administered at Shandong Cancer Hospital. The inclusion criteria are as follows: Patients must be aged 18 to 80 years and have histologically confirmed urothelial carcinoma with measurable lesions according to Response Evaluation Criteria in Solid Tumors (RECIST) Version 1.1, along with an Eastern Cooperative Oncology Group (ECOG) performance status score of ≤2. The study includes individuals with locally advanced unresectable or metastatic urothelial carcinoma originating from the renal pelvis, ureter, bladder, or urethra, as confirmed by histopathological or cytological analysis. This encompasses both transitional cell carcinoma and mixed subtypes involving transitional and non-transitional cell carcinoma. The criteria for platinum intolerance must meet at lea8st one of the following conditions: An ECOG performance status of 2, renal impairment with a creatinine clearance between 30 and 60 mL/min, sensorineural hearing loss of grade ≥2, peripheral neuropathy of grade ≥2, or New York Heart Association (NYHA) Class II heart failure; no previous systemic chemotherapy for advanced stage disease; perioperative platinum-based chemotherapy was allowed if disease recurrence occurred over 12 months after treatment completion; Sufficient baseline organ function, including normal hematological parameters (absolute neutrophil count, platelet count, and hemoglobin concentration), renal function (assessed via creatinine clearance rate), and hepatic function (evaluated by total bilirubin, alanine aminotransferase, and aspartate aminotransferase levels); The life expectancy must be more than 3 months as estimated by the investigator at enrollment.

The exclusion criteria are as follows: active central nervous system (CNS) metastases or carcinomatous meningitis; definitive diagnosis of autoimmune diseases or interstitial lung disease; persistent systemic infections requiring therapeutic intervention; inadequately managed diabetes mellitus, characterized by glycated hemoglobin (HbA1c) levels of ≥8% or levels between 7% and <8% in conjunction with unexplained polyuria or polydipsia; active infections with hepatitis B virus (HBV), hepatitis C virus (HCV), or human immunodeficiency virus (HIV); imminent spinal cord compression; or prior exposure to therapies targeting T-cell costimulatory or checkpoint pathways. Moreover, the use of any anticancer monoclonal antibodies was strictly prohibited within the four-week period preceding the commencement of the trial.

### 2.2 Procedures

Patients were administered tislelizumab at a standardized dosage of 200mg via intravenous infusion on day 0. Gemcitabine was delivered intravenously at a dose of 1.0 g/m^2^ on days 1 and 8 of each 21-day treatment cycle.

The evaluation of treatment responses, including complete response (CR), partial response (PR), stable disease (SD), and progressive disease (PD), was determined in accordance with the RECIST version 1.1 guideline.

Patients who were evaluated as CR, PR, or SD in the 4–6 cycles efficacy assessment can choose tislelizumab for maintenance treatment. Treatment continued until any of the following occurred: confirmed disease progression, intolerable toxicity, withdrawal by the physician or patient, an intercurrent illness precluding further therapy, non-adherence to trial protocol, lost contact and could not be followed up, or the 24-month treatment was finished. Dose reduction was not permissible for tislelizumab. Gemcitabine dosage was modified in accordance with predefined guidelines to manage treatment-emergent adverse events (AEs). Post-progression treatment was not mandated by the protocol and was selected at the investigator’s discretion according to patient condition, biomarker status, drug accessibility, and local practice.

The assessment of treatment efficacy was performed using either computed tomography (CT) or magnetic resonance imaging (MRI), with MRI serving as the preferred imaging modality for patients with renal impairment who were unable to tolerate contrast-enhanced CT scans. Imaging evaluations were planned every 8 weeks (range:7–9 weeks) during the initial 56 weeks of therapy, and subsequently conducted once every 12 weeks (range:11–13 weeks). The main endpoint of objective response was established through radiographic evidence, with initial response findings requiring confirmation via scan of a follow-up performed no less than 4 weeks from the beginning assessment or during the next at the subsequent scheduled imaging visit. Similarly, suspected radiological disease progression necessitated verification through a repeat scan obtained a minimum of 4 weeks later. Notably, investigators were authorized to continue tislelizumab administration for patients who exhibited radiologically confirmed PD but maintained clinical stability and demonstrated clinical benefit from treatment, until the occurrence of further disease progression. CR and PR were confirmed at least 4 weeks following the initial documentation by investigators via repeat imaging.

All AEs were documented from the first day of treatment until 30 days post-treatment cessation, with serious adverse events (SAEs) being monitored for an extended period of 90 days post-treatment. AEs were categorized and assessed according to the National Cancer Institute Common Terminology Criteria for Adverse Events (CTCAE) Version 5.0.

Safety surveillance procedures, including physical assessments and laboratory analysis (hematological as well as clinical chemistry panels), were systematically conducted on all days involving drug administration. Baseline laboratory assessments — including hematology, clinical chemistry, and urinalysis — were completed within 10 days prior to treatment initiation; subsequent tests were performed within 72 hours before each dose administration starting from cycle 2.

Patients who experienced grade 4 treatment-related AEs need permanently cease tislelizumab therapy. In cases of grade 3 treatment-related AEs, therapy was temporarily halted until the toxicities diminished to grade 1 or below; Treatment was permanently stopped if such resolution was not achieved within 12 weeks after the last tislelizumab dose. Furthermore, the permanent discontinuation of treatment was mandated for certain grade 3 treatment-associated events, such as type 1 diabetes mellitus, infusion-related reactions, pneumonitis, renal failure or nephritis, and clinically noteworthy increases in aspartate aminotransferase, alanine aminotransferase, or bilirubin concentrations.

For biomarker analysis, tumor tissue specimens underwent routine pathological evaluation. Upon enrollment, each patient was required to provide 10 formalin-fixed, paraffin-embedded (FFPE) tumor biopsy sections mounted on charged slides. These samples underwent immunohistochemistry (IHC) and dual-plex immunofluorescence (IF) staining for the characterization of TLS.

Although PD-L1 expression levels were not used as a patient enrollment criterion and submission of relevant specimens was not mandatory, the assessment of PD-L1expression was conducted on baseline archival or recently acquired tumor samples through a validated 22C3 PD-L1 IHC assay. A certified pathologist evaluated the immunostained slides employing two methods: the H-score system (spanning from 0 to 300) and the combined positive score (CPS), with PD-L1 expression stratified into two categories (low: CPS < 10; high: CPS ≥ 10).

### 2.3 Outcomes

The study’s primary outcome measures encompassed the ORR and disease control rate (DCR), while the secondary outcome parameters included PFS and OS, and treatment-related adverse events (TRAEs). The term ORR denotes the percentage of patients achieving either a complete response (CR) or a partial response (PR). The DCR was determined as the proportion of patients exhibiting CR, PR, or SD, based on investigator evaluation. In accordance with the RECIST version 1.1, PFS was assessed from the commencement of treatment until the initial documented disease progression, death from any cause, or discontinuation of therapy at the patient’s request. OS was defined as the interval from the first dose administration to all-cause death. This study analyzed TRAE using the CTCAE version 5.0.

### 2.4 TLS detection

Our investigation centered on identifying TLS. The detection of TLS in tumor samples was carried out using chromogenic immunohistochemistry (IHC) and two-plex immunofluorescence (IF). Preliminary assessment of hematoxylin and eosin (H&E)-stained sections demonstrated round or oval lymphocyte aggregates in the tumor stroma, along with peritumoral and intratumoral areas. Subsequently, IHC was employed for definitive TLS confirmation based on the co-expression of specific markers: CD20+ B cells, CD3+/CD4+/CD8+ T cells, and CD21+/CD23+ follicular dendritic cells. Any inconsistencies in interpretation were adjudicated via consensus review.

The EnVision detection system (Dako) was employed to visualize CD3, CD4, CD8, CD20, CD21, and CD23 markers following processing on an Autostainer system (Agilent Technologies). An experienced pathologist utilized HALO 10 image analysis software (Indica Labs) to outline tumor regions, guided by reference slides stained with hematoxylin and eosin. A specialized integrated classifier was applied to identify TLS. Within the delineated tumor and TLS areas, the quantities of CD3+, CD4+, CD8+, CD20+, CD21+, and CD23+ cells were assessed and integrated into a dedicated testing platform. We referred to the International Immuno-Oncology Biomarkers Working Group to confirm the specific testing methods of TLS in UC (18).

### 2.5 Statistical analysis

Statistical analyses were performed using SPSS (Version 27.0; IBM Corp.) and R (Version 4.5.0) software packages. Categorical variables were analyzed utilizing the chi-squared test or Fisher’s exact test, continuous variables were evaluated through independent-sample t-tests or nonparametric rank-sum tests. Kaplan-Meier (K-M) methodology was utilized to estimate OS and PFS. Cox proportional-hazards regression models produced hazard ratios (HRs) along with corresponding 95% confidence intervals (CIs). The threshold for statistical significance was established at P ≤ 0.05. The clinical and demographic attributes of the patient cohort were delineated through the application of descriptive statistical methods. Due to the small sample size and limited number of events, multivariable Cox proportional hazards regression or logistic regression analyses were not performed to avoid overfitting. Sensitivity analyses were not pre-planned given the descriptive, single-arm design of the study. All efficacy and safety analyses were conducted in the full analysis set, defined as all enrolled patients who received at least one dose of study treatment.

## 3 Results

### 3.1 Patient characteristics

**Figure 1:**
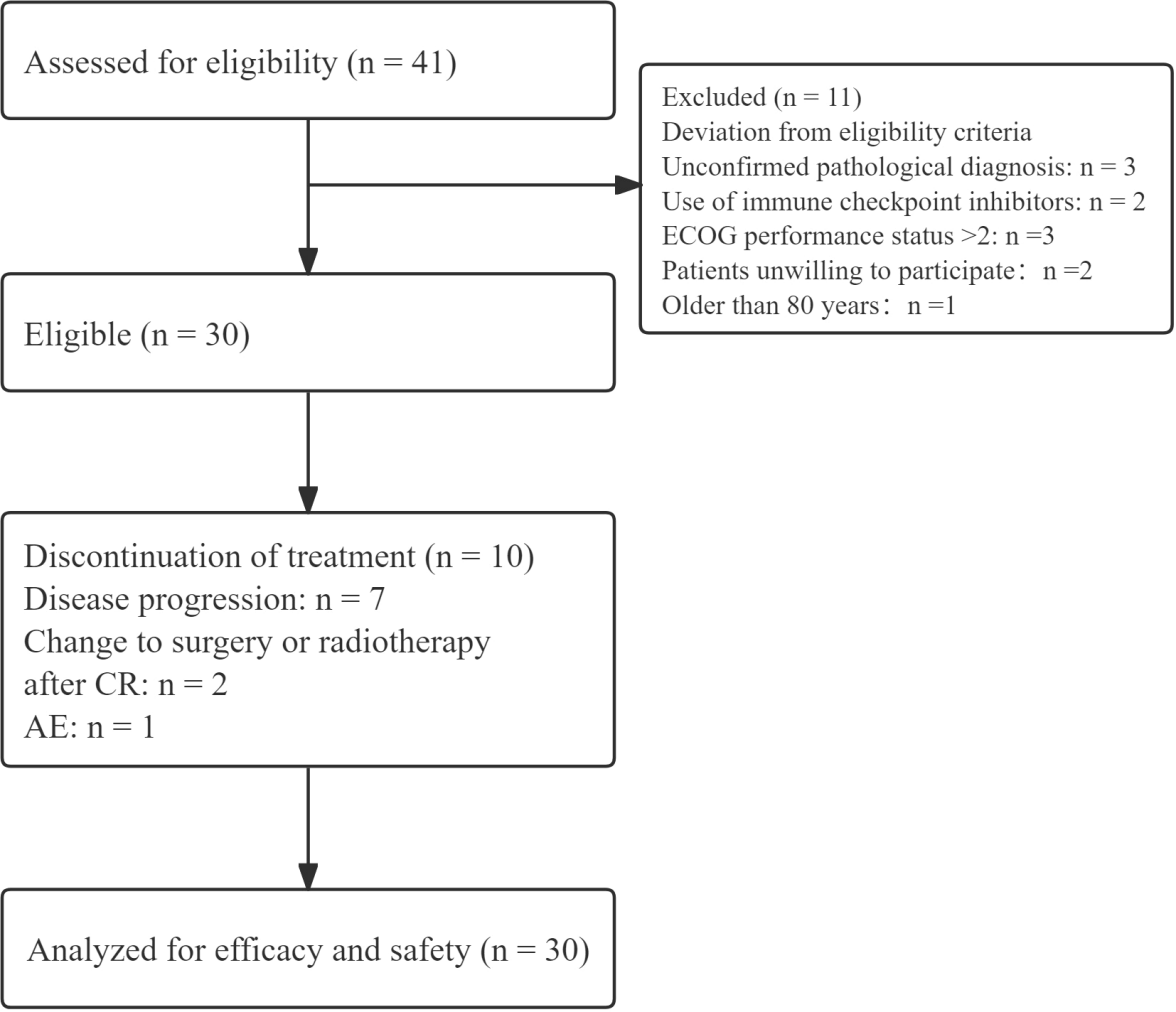
Research flowchart. A total of 41 patients were screened, among whom 30 met the inclusion criteria and received treatment, and finally underwent efficacy and safety analyses.

From July 2, 2022, to December 1, 2024, a total of 41 patients underwent screening at Shandong Cancer Hospital. Of this cohort, 30 patients who met the inclusion criteria were enrolled and deemed eligible for subsequent statistical analysis (**Figure 1**). **Table 1** presents the general characteristics of the patients in the study. The patients’ median age was 67 years, ranging from 40 to 79 years. The study population exhibited a predominance of male patients (70.0%) and individuals younger than 75 years of age (76.7%). Urothelial carcinoma constituted the predominant pathological subtype, accounting for 90.0% of all cases. Additionally, 76.7% of patients had an ECOG PS score of 1, 60% displayed PD-L1 expression levels of ≥1%.

**Table 1:** Patient baseline clinical characteristics.

Characteristics	Cohort (n=30)
Sex	
Female	9 (30.0%)
Male	21 (70.0%)
Age	
Median	67 (40–79)
< 75	23 (76.7%)
≥ 75	7 (23.3%)
ECOG	
0	5 (16.7%)
1	23 (76.7%)
2	2 (6.7%)
Renal function based on creatinine clearance, mL/min	
Normal: ≥90	17 (56.7%)
Mild decrease: ≥60 and <90	11 (36.7%)
Moderate decrease: ≥30 and <60	2 (6.7%)
Primary tumor location	
Upper tract (renal pelvis and ureter)	8 (26.7%)
Bladder or other	22 (73.3%)
Histology type	
Urothelial carcinoma only	27 (90.0%)
Mixed Urothelial	3 (10.0%)
Metastasis sites	
≥3	15 (50.0%)
<3	15 (50.0%)
Liver metastasis	
Yes	7 (23.3%)
No	23 (76.7%)
Single Kidney	
Yes	8 (26.7%)
No	22 (73.3%)
PD-L1 expression	
<1%	7 (23.3%)
≥1%	18 (60.0%)
Unknown	5 (16.7%)
TLS Expression Levels	
TLS-positive	9 (30.0%)
TLS-negative	17 (56.7%)
Unknown	4 (13.3%)

ECOG, Eastern Cooperative Oncology Grou; PD-L1, programmed-death ligand 1; TLS, tertiary lymphoid structures.

### 3.2 Efficacy

**Figure 2:**
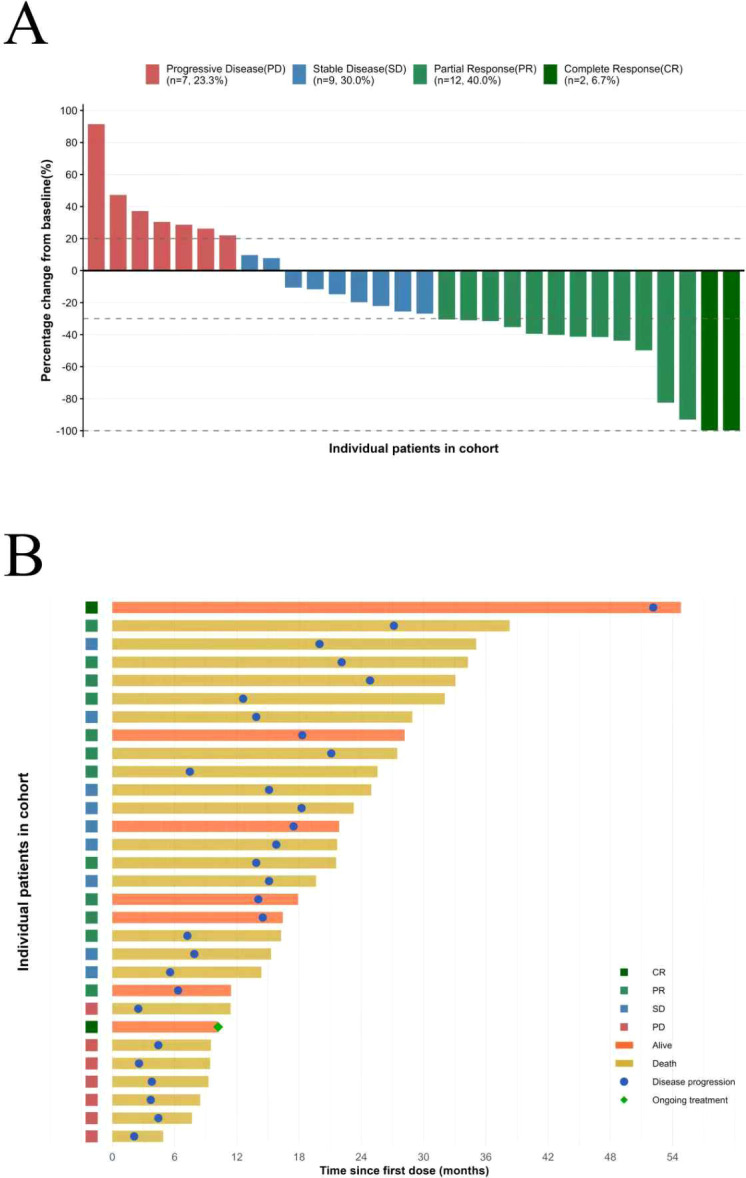
Change in target lesions from baseline **(A)** and responses in patients with a confirmed objective response **(B)**. **(A)** The maximum percentage change of the target lesion relative to the baseline. The same therapy group used the same color for classification. **(B)** Swimmers plot of time-to-response and duration. Seven patients remained alive and one patient continued to receive treatment.

**Figure 3:**
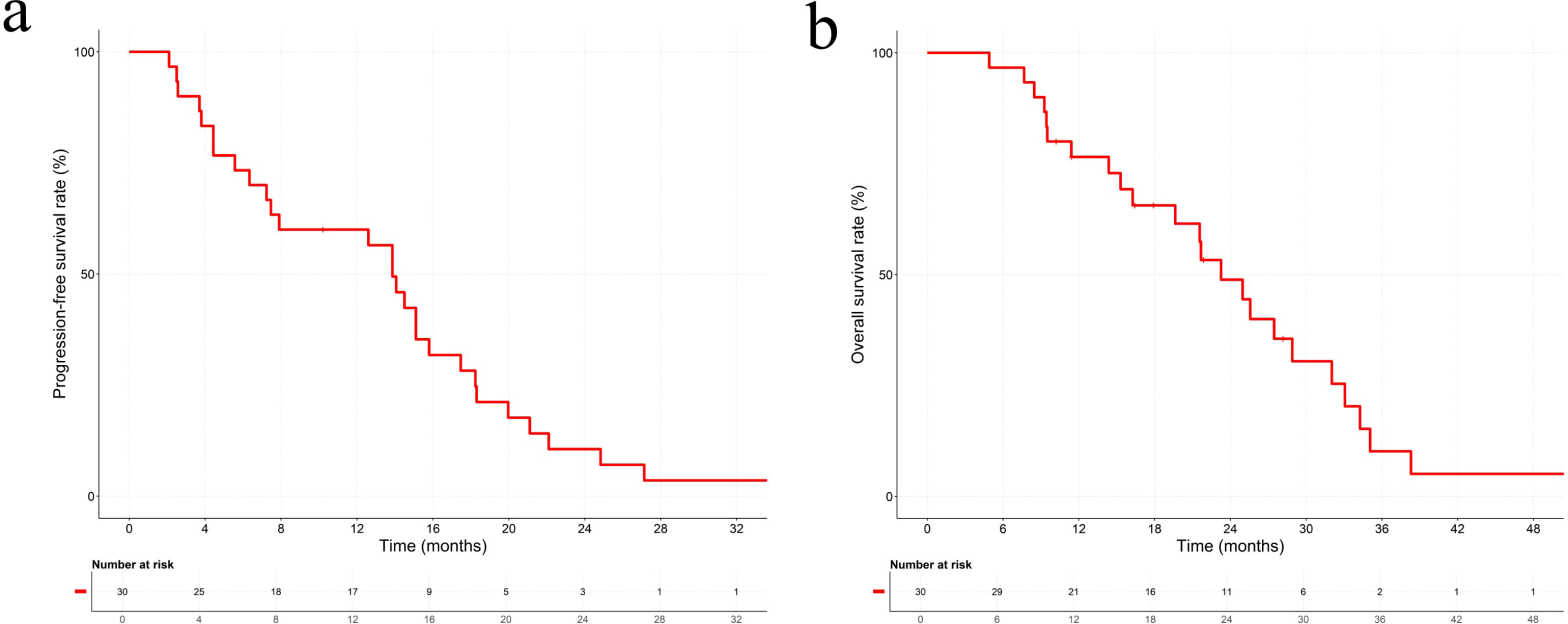
Progression-free survival **(a)** and overall survival **(b)**.

As detailed in **Table 2**, the confirmed ORR was 46.7% (14/30; 95% confidence interval [CI]: 28.3–65.7), while and DCR reached 76.7%. **Figure 2A** illustrates the maximum percentage reduction in target lesion size relative to baseline measurements. Specifically, 2 patients (6.7%) achieved CR and 12 patients (40%) attained PR, as depicted in **Figure 2B**. The median PFS was 13.9 months (95% CI: 11.4–16.3; **Figure 3a**), and the median OS was 23.3 months [interquartile range (IQR): 14.4–33.1; 95% CI: 18.2–28.3; **Figure 3b**). Among the 14 responders, the median time to response was 1.92 months (range,1.51–3.12 months]. The median duration of response (DOR) estimated using the Kaplan-Meier method was 12.22 months (95% CI, 4.93–19.22 months). A total of 23 (CR: 2; PR: 12 and SD: 9) patients entered into maintenance therapy after receiving 4–6 cycles of gemcitabine combined with immunotherapy. Furthermore, on the cutoff date of September 1, 2025, seven patients were still alive, one patient was still undergoing treatment.

**Table 2:** Objective response, and disease control rate.

Efficacy evaluation	Cohort (n=30)
ORR	14 (46.7%)
95% CI	28.3–65.7
DCR	23 (76.7%)
CR	2 (6.7%)
PR	12 (40.0%)
SD	9 (30.0%)

ORR, Objective response rate; CR, Complete response; PR, partial response; SD, Stable disease; DCR, disease control rate.

### 3.3 Safety

The overall safety profile of the treatment regimen is presented in **Table 3**. Any-grade TRAEs were observed in 73.3% of the participants involved in the study. The predominant TRAEs were hematological toxicities, with neutropenia occurring in 12 patients (40.0%) and leukopenia in 13 patients (43.3%). Severe adverse events (Grade 3-4) were observed in 4 patients (13.3%), encompassing fatigue (3.3%), decreased appetite (3.3%), nausea (3.3%), elevated alanine transaminase (ALT) levels (3.3%), and myelosuppression (10%). TRAEs resulted in gemcitabine dose reduction for two patients due to grade 3–4 myelosuppression. One patient discontinued treatment following four cycles due to grade 3 fatigue. No treatment-related mortality was recorded during the study period.

**Table 3:** Treatment-related adverse events.

Adverse event	Any grade n (%)	Grade 1–2 n (%)	Grade 3 n (%)	Grade 4 n (%)
Any events	22 (73.3%)	18 (60.0%)	3 (10.0%)	1 (3.3%)
Event leading to discontinuation of treatment	1 (3.3%)	—	1 (3.3%)	—
Fatigue	5 (16.7%)	4 (13.3%)	1 (3.3%)	—
Stomatitis	2 (6.7%)	2 (6.7%)	—	—
Decreased appetite	6 (20.0%)	5 (16.7%)	1 (3.3%)	—
Nausea	5 (16.7%)	4 (13.3%)	1 (3.3%)	—
Diarrhea	1 (3.3%)	1 (3.3%)	—	—
Rash	2 (6.7%)	2 (6.7%)	—	—
Fever	1 (3.3%)	1 (3.3%)	—	—
Anemia	12 (40.0%)	10 (33.3%)	2 (3.3%)	—
Leukopenia	13 (43.3%)	10 (33.3%)	2 (3.3%)	1 (3.3%)
Neutropenia	12 (40.0%)	9 (30.0%)	2 (3.3%)	1 (3.3%)
Thrombocytopenia	7 (23.3%)	4 (13.3%)	2 (3.3%)	1 (3.3%)
Hypercholesteremia	1 (3.3%)	1 (3.3%)	—	—
ALT increased	3 (10.0%)	2 (6.7%)	1 (3.3%)	—
AST increased	3 (10.0%)	3 (10.0%)	—	—
Bilirubin increase	7 (23.3%)	7 (23.3%)	—	—
Adrenal insufficiency	1 (3.3%)	1 (3.3%)	—	—
Elevated Creatinine	2 (6.7%)	2 (6.7%)	—	—
Hyperuricemia	4 (13.3%)	4 (13.3%)	—	—
Thyroid Dysfunction	4 (13.3%)	4 (13.3%)	—	—
Myalgia	1 (3.3%)	1 (3.3%)	—	—
Thromboembolic events	5 (16.7%)	5 (16.7%)	—	—
Urinary tract infection	2 (6.7%)	2 (6.7%)	—	—
Cardiac troponin T increased	4 (13.3%)	4 (13.3%)	—	—
Electrocardiogram T wave changes	6 (20.0%)	6 (20.0%)	—	—

### 3.4 Exploratory analysis of TLS

**Figure 4:**
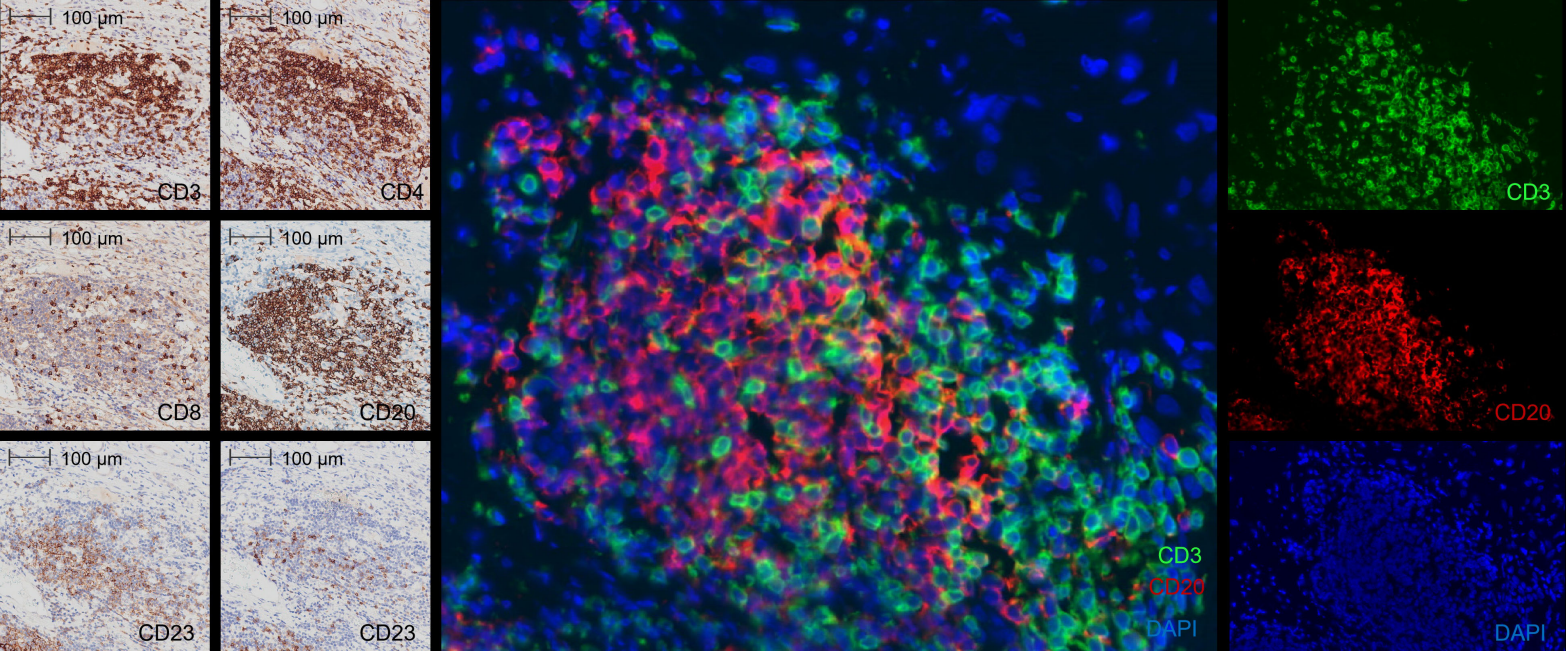
Characteristic of the composition of TLS. IHC staining for CD3, CD4, CD8, CD20, CD21 and CD23 (original magnification×20), and IF staining of the same region of CD3 and CD20, CD20 (in red) and CD3 (in green) show congruent localization. Nuclei were counterstained with DAPI (blue). The figure illustrates the spatial arrangement of B cells at the center surrounded by T cells within the TLS.

Among the 30 enrolled patients, 26 patients participated in the analysis of TLS (**Figure 4**). There was a quantitative difference in ORR according to the results of TLS analysis, with response rates of 66.7% in TLS positive group (n=9) and 35.3% in TLS negative group (n=17) respectively. However, this observed change did not reach statistical significance (P = 0.218).

## 4 Discussion

The management of platinum-ineligible patients represents a significant clinical challenge in advanced urothelial carcinoma. Among cisplatin-unfit individuals receiving carboplatin-based therapy, approximately 15% experience poor outcomes, with low response rates and suboptimal tolerability, especially in those with renal impairment (19, 20).

Data from the KEYNOTE-052 trial indicated that renal dysfunction accounted for cisplatin ineligibility in about 59% of cases. Patients with diminished renal function have demonstrated a more favorable safety profile when treated with PD-1/PD-L1 inhibitors. Treatment responses were observed across diverse subgroups, including age, tumor site, ECOG performance status, liver metastasis status, and specific reasons for cisplatin ineligibility, highlighting the broad activity of PD-1 inhibitors in cisplatin-ineligible populations (21). The results of IMvigor210 provide additional evidence supporting the application of immunotherapy in patients who cannot receive cisplatin-containing regimen, as renal failure was observed in only 2% of the enrolled individuals with advanced urothelial cancer (11). Consequently, these agents represent a suitable therapeutic option even for patients who might otherwise be limited to best supportive care.

In the current treatment guidelines, EV plus ICI have shown pronounced efficacy in la/mUC (23). Based on the results of EV-302 trial, the FDA approved this combination for previously untreated advanced urothelial cancer, including cisplatin-ineligible cases. Consequently, both the National Comprehensive Cancer Network (NCCN) and international guidelines now endorse EV combined with pembrolizumab as a recommended initial therapeutic regimen (24, 25). ADC drugs indeed have excellent efficacy in la/mUC, regardless of whether platinum tolerance or intolerance. However, it is worth noting that in EV-302 trial, the incidence of peripheral sensory neuropathy in EV group (all grades 50%; grade ≥ 3 3.6%) was significantly higher compared to the chemotherapy group (all grades 9.9%). Besides, due to limitations in accessibility and cost, the actual use of these drugs is fraught with numerous difficulties. Therefore, it is necessary to investigate alternative strategies to resolve this urgent problem.

In our study, platinum was removed from the traditional first-line treatment regimen, and gemcitabine, which has relatively low nephrotoxicity, was selected for combination with immunotherapy. These results have potential clinical significance.

In KEYNOTE-052 trial, pembrolizumab was used in cisplatin-ineligible la/mUC patients as initial treatment, the study reported an ORR of 28.6%, a median duration of response (DOR) extending to 30.1 months, and a median OS of 11.3 months. The most common grade 3–4 treatment-related AE were fatigue (2%), alkaline phosphatase increase (1%), colitis, and muscle weakness (both 1%), and 36 (10%) patients had a serious TRAE (10).

Another independent comparative analysis assessed the efficacy of a PD -1 inhibitor in contrast to carboplatin-gemcitabine chemotherapy as an initial therapeutic approach for cisplatin-ineligible patients with advanced upper tract urothelial carcinoma. The ORR were similar between the PD-1 inhibitor and chemotherapy arms (38.6% vs. 41.5%). In the PD-1 inhibitor group, the median PFS was 5.0 months and OS was 18.0 months, whereas in the carboplatin-gemcitabine group, these values were 7.0 months and 14.0 months, respectively (P = 0.166; P = 0.257). The incidence of adverse reactions related to immune checkpoint inhibitors therapy is lower (57.1% compared to 73.3%) (22).

In our study, the confirmed ORR was 46.7% (14 of 30 patients; 95% CI: 28.3-65.7), DCR was 76.7%, with 2(6.7%) achieving a CR and 12 (40%) achieving PR. The mPFS was 13.9 months (95% CI: 11.4–16.3), and the mOS was 23.3 months (95% CI: 18.2–28.3). The integration of tislelizumab with gemcitabine demonstrated a tolerable safety profile among patients ineligible for cisplatin, accompanied by a similar incidence of grade 3–4 TRAEs (13.3%) compared with pembrolizumab alone (lower than 16%). And the most common TRAEs were hematological toxicities. Treatment cessation occurred in a solitary instance as a result of grade 3 fatigue (3.3%). No deaths associated with the treatment were documented. The safety results obtained in this analysis are consistent with the findings documented in prior research.

Prespecified subgroup analyses of ORR by age, sex, primary tumor location, metastasis sites, histology type, liver metastasis, PD-L1 expression and TLS expression levels showed some differences. The female patients had a higher response rate than male patient. In addition, patients without liver metastasis had a higher ORR. Moreover, we observed that tumors of other pathological types (except urothelial carcinoma) were not responsive to the treatment, and the prognosis is poor.The epidemiology and causes of UTUC in Chinese patients differ from those in Western populations. In China, the incidence of UTUC is higher (range, 9.3%–29.9%), partly because of exposure to aristolochic acid (AA) (26, 27). In Western populations, UTUC is relatively uncommon, accounting for only 5%–10% of urothelial carcinoma cases (28). The upper tract (renal pelvis and ureter) patients account for 26.7% (8/30) in our study. Our trial protocol has shown efficacy across all sites of urothelial carcinoma, with a slightly higher response rate for upper urinary tract patients (50% vs 46%). It has definite therapeutic effects and relatively low side effects, making it a reasonable option for patients who are intolerant to platinum.

The relationship between TLS and immunotherapy as well as prognosis has also been explored in urothelial carcinoma (29, 30). The current study undertook an exploratory assessment of TLS in relation to treatment efficacy. Although the ORR was numerically higher in the TLS-positive group than in the TLS-negative group (66.7% vs. 35.3%), this difference did not reach statistical significance (P = 0.218). Based on the current data, the evidence is insufficient to regard TLS as a validated predictive indicator for antitumor treatment efficacy, and further investigation is warranted.

Our study had several limitations. First, due to the fact that this study is a single center, single arm clinical trial, comparative analysis was not feasible. Second, due to the limited sample size, only 86.7% (26 out of 30) of the samples could be utilized for testing TLS expression, although the positive group showed better efficacy than the negative group numerically speaking (66.7% versus 35.3%), there is no statistical difference between the two sets of data analysis (P = 0.218). Third, while owing to the modest sample size, multivariable analyses were not conducted to prevent overfitting, and formal sensitivity analyses were not performed. Subgroup analyses (**Table 4**) were instead used to explore potential heterogeneity. Fourth, the relatively favorable baseline characteristics, including the predominance of ECOG PS 0–1 and the limited proportion of patients with liver metastasis, together with the small sample size, may have contributed to the observed median OS and may limit the generalizability of the findings. Fifth, PD-L1 expression was not used for patient selection, and PD-L1 subgroup analyses were descriptive only; this study was not powered to assess associations between PD-L1 expression and survival outcomes. Finally, post-progression treatment data were not systematically quantified in this manuscript, so the potential influence of subsequent therapies on OS cannot be excluded. These statistical and clinical limitations should be considered, and further validation in randomized trials is required.

**Table 4:** Subgroup analysis of objective response rate.

Characteristics	n/N	ORR (95% CI)
Sex		
Male	8/21	38% (18.1-61.6)
Female	6/9	67% (29.9-92.5)
Age		
≥ 75	4/7	57% (18.4-90.1)
< 75	10/23	44% (23.2-65.5)
Primary tumor location		
Upper tract (renal pelvis and ureter)	4/8	50% (15.7-84.3)
Bladder or other	10/22	46% (24.4-67.8)
Histology type		
Urothelial carcinoma only	14/27	52% (31.9-71.3)
Mixed Urothelial	0/3	0% (0.0-70.8)
Metastasis sites		
≥3	6/15	40% (16.3-67.7)
<3	8/15	53% (26.6-78.7)
Liver metastasis		
Yes	2/7	29% (3.7-71.0)
No	12/23	52% (30.6-73.2)
PD-L1 expression		
<1%	2/7	29% (3.7-71.0)
≥1%	10/18	56% (30.8-78.5)
Unknown	2/5	40% (5.3-85.3)
TLS Expression Levels		
TLS-positive	6/9	67% (29.9-92.5)
TLS-negative	6/17	35% (14.2-61.7)
Unknown	2/4	50% (6.8-93.2)

In summary, we conduct a single-center, single-arm, phase 2 research to explore the efficacy and tolerability of combining tislelizumab with gemcitabine as an initial treatment for patients with la/mUC who are ineligible for cisplatin-containing treatment. The findings from our dataset indicate that the tislelizumab-gemcitabine combination displayed a promising preliminary efficacy signal alongside a manageable safety profile in this specific cohort of patients. Based on our current understanding, this is the first report detailing the application of this therapeutic strategy in this specific cohort. The promising results could potentially offer a new and feasible treatment alternative for cisplatin-ineligible urothelial carcinoma patients and require confirmation in larger randomized controlled trials.

## Data Availability

The original contributions presented in the study are included in the article/supplementary material. Further inquiries can be directed to the corresponding author/s.
